# Clinically integrated multi-modal transformer framework with cross-modal gated fusion and clinical nomogram for automated Kellgren-Lawrence grading of knee osteoarthritis on x-ray images

**DOI:** 10.1186/s12891-026-10043-4

**Published:** 2026-06-15

**Authors:** Yingying Huang, Zihan Shao, Renfang Wang, Hong Qiu

**Affiliations:** 1 College of Big Data and Software Engineering, Zhejiang Wanli University, No. 8 Qianhu South Road, Ningbo, Zhejiang 315100 China; 2 School of Informatics, The University of Edinburgh, Edinburgh, UK

**Keywords:** Knee osteoarthritis, Kellgren-Lawrence grading, Multi-modal Transformer, Cross-modal fusion, Clinical nomogram, External validation, Uncertainty quantification

## Abstract

**Background:**

We developed a multi-modal Transformer framework integrating knee radiographs with clinical covariates to enable automated, objective, and generalizable ordinal Kellgren-Lawrence (KL) grading.

**Methods:**

A total of 2,703 anteroposterior knee radiographs were retrospectively collected from three independent medical centers (January 2018 - December 2024). Data from two centers (*n* = 1,953) were used for model development and internal five-fold stratified cross-validation, while the third center (*n* = 750) served as an independent external test set. The proposed framework combines a Swin Transformer-Base image encoder with a clinical feature Transformer through a novel Robust Cross-Modal Gated Fusion (RCGF) module employing bidirectional cross-attention and uncertainty-aware dynamic gating via Monte-Carlo dropout. Ordinal prediction was performed using Consistent Rank Logits (CORAL). Eight classifier architectures were systematically compared, encompassing multi-modal models, unimodal image-only baselines, and a clinical-only model.

**Results:**

The proposed RCGF framework achieved a Quadratic Weighted Kappa (QWK) of 0.900 (95% CI: 0.877–0.921), macro-averaged AUC of 0.930 (95% CI: 0.910–0.950), and balanced accuracy of 87.6% on the independent external test set, significantly outperforming all baseline models including BioViL-T (QWK = 0.850) and MedViT (QWK = 0.830; all FDR-corrected *p* < 0.001). Sensitivity for severe Osteoarthritis (OA) (Grade 4) reached 83.5% (95% CI: 79.1–87.4%), with specificity 95.3%. The clinical nomogram demonstrated excellent calibration (calibration slope = 0.98, Brier score = 0.072, C-statistic = 0.940) and superior net benefit over treat-all and treat-none strategies across all clinically relevant decision thresholds.

**Conclusion:**

This multi-modal Transformer framework with uncertainty-aware gated fusion provides robust external generalizability for ordinal knee OA severity grading and delivers a clinically actionable nomogram. The approach has strong potential to reduce radiologist workload and facilitate objective assessment on routine clinical radiographs, particularly in resource-constrained settings.

**Supplementary Information:**

The online version contains supplementary material available at 10.1186/s12891-026-10043-4.

## Introduction

Knee osteoarthritis (OA) represents the most prevalent joint disease worldwide and a major cause of chronic pain, disability, and reduced quality of life. It affects over 500 million individuals globally, with radiographic evidence present in approximately 37% of women and 27% of men aged over 60 years [1]. The socioeconomic burden is enormous, with direct and indirect costs exceeding $200 billion annually in high-income countries, projected to rise sharply with population aging [2]. Accurate severity assessment is critical for risk stratification, treatment planning, and monitoring disease progression.

The current gold standard, the Kellgren-Lawrence (KL) grading system, is semi-quantitative, suffers from substantial inter-observer variability (Cohen’s κ = 0.45–0.65 in community radiological settings), and depends heavily on the expertise of musculoskeletal radiologists [3, 4]. This variability imposes a direct clinical cost: patients in settings without specialist coverage may receive suboptimal or delayed care.

Although deep learning approaches have shown promise in automating knee radiograph interpretation [5–7]. several important limitations persist. Most models perform binary classification (OA versus non-OA) rather than full ordinal grading across the five KL levels, losing clinically relevant severity information [5, 8]. The majority of published studies rely on single-center data with only internal validation, limiting generalizability across heterogeneous imaging protocols, scanner vendors, and patient populations [9, 10].Critically, most existing frameworks are unimodal (image-only) and fail to incorporate readily available clinical risk factors [11, 12]. Even when multi-modal approaches are attempted, they typically rely on simple concatenation or late-fusion strategies that lack robustness to domain shift and fail to model uncertainty in feature integration [6, 13].

To overcome these limitations, we propose a novel multi-modal Transformer-based framework that integrates high-resolution knee radiographs with key clinical covariates through a Robust Cross-Modal Gated Fusion (RCGF) module. Unlike conventional fusion methods, RCGF employs bidirectional cross-attention combined with an uncertainty-aware dynamic gating mechanism based on Monte-Carlo dropout, enabling adaptive weighting of each modality while explicitly addressing inter-center domain shift. Ordinal prediction is achieved using the Consistent Rank Logits (CORAL) framework to preserve the natural ordering of KL grades [8]. The model was developed and internally validated using five-fold cross-validation on data from two medical centers (*n* = 1,953) and externally tested on an independent third center (*n* = 750).

While Swin Transformer, CORAL, MC-Dropout, and clinical feature fusion are individually established methods, their principled integration into a unified uncertainty-aware cross-modal gated fusion framework for ordinal knee OA grading, combined with multi-center external validation and a validated clinical nomogram, has not been previously reported. The novelty of this work lies in this specific combination and its rigorous clinical validation, rather than in any single component.

## Methods

**Fig. 1:**
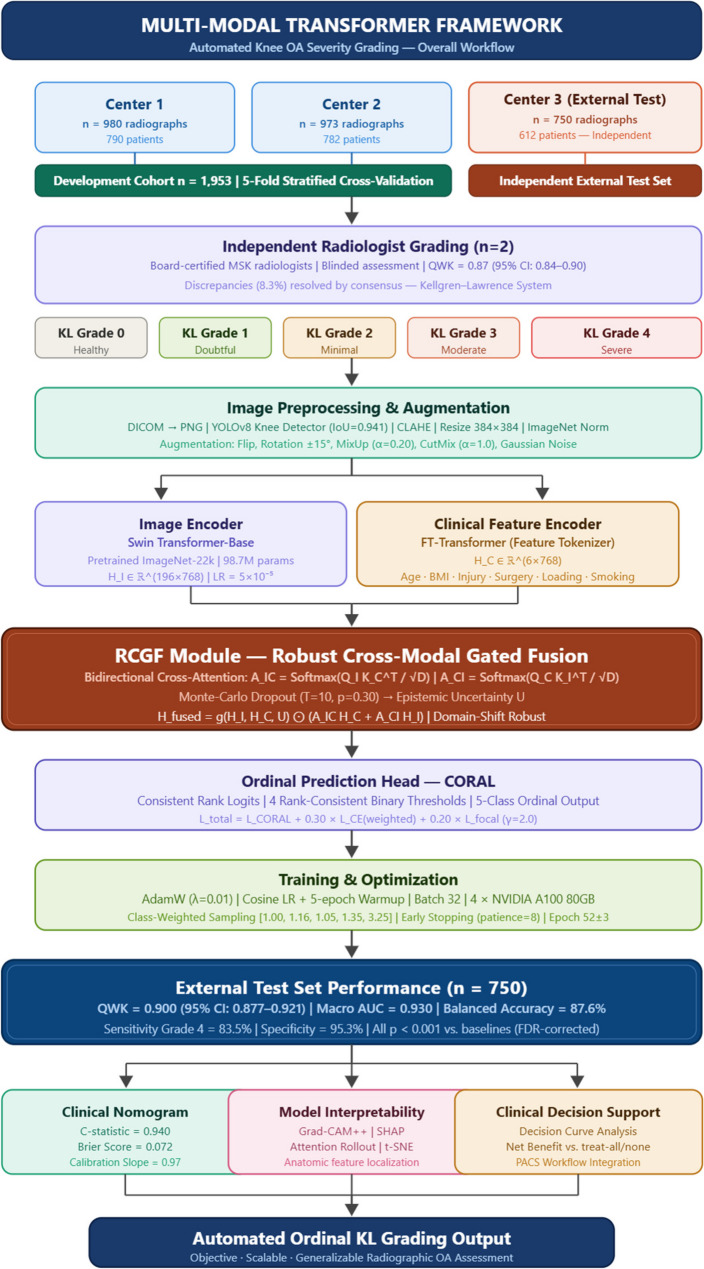
Overall workflow of the proposed multi-modal transformer framework, illustrating the pipeline from multi-center data collection and image preprocessing through the Robust Cross-Modal Gated Fusion (RCGF) module, ordinal CORAL prediction, and clinical nomogram construction. Arrows indicate data flow between components

Figure 1 illustrates the overall workflow of the proposed multi-modal Transformer framework, including multi-center data collection, image preprocessing, the Robust Cross-Modal Gated Fusion (RCGF) module, ordinal CORAL prediction, clinical nomogram construction, and model interpretability tools.

### Study population and dataset

This retrospective multi-center study was approved by the institutional review boards of all three participating centers, with a waiver of informed consent due to the de-identified nature of the data. The study was conducted and reported in accordance with the TRIPOD + AI guidelines for transparent reporting of a multivariable prediction model for individual prognosis or diagnosis. The study protocol was approved by the Institutional Review Board of Zhejiang Wanli University (approval no. 62576319), with a waiver of informed consent due to the fully de-identified nature of the data.

A total of 2,703 anteroposterior knee radiographs were collected from three independent medical centers between January 2018 and December 2024. Center 1 contributed 980 radiographs (790 patients) and Center 2 contributed 973 radiographs (782 patients); these two centers (combined *n* = 1,953 from 1,572 patients) constituted the development cohort used for five-fold stratified cross-validation. Center 3 (*n* = 750 from 612 patients) served as an independent external test set with no overlap with the development cohort. Detailed imaging acquisition parameters for each center are provided in Supplementary Table S2.

Inclusion criteria were: age ≥ 40 years, standard weight-bearing AP knee radiograph of diagnostic quality, and complete availability of all six predefined clinical variables. Exclusion criteria included: previous total or unicompartmental knee arthroplasty, acute fracture, primary or metastatic bone tumor, septic arthritis, severe motion artifact, or incomplete clinical records. In total, 247 radiographs were excluded (Center 1: 83, Center 2: 91, Center 3: 73), primarily due to motion artifact (*n* = 104) and incomplete clinical data (*n* = 87).

Knee OA severity was graded independently by two board-certified musculoskeletal radiologists (Radiologist A: 14 years of experience; Radiologist B: 11 years of experience) using the standardized Kellgren-Lawrence (KL) system, blinded to clinical information and each other’s assessments. Inter-observer agreement was substantial (Quadratic Weighted Kappa = 0.87; 95% CI: 0.84–0.90; intraclass correlation coefficient = 0.91). Discrepancies (occurring in 8.3% of cases) were resolved by consensus at a joint review session. KL grade definitions followed published criteria: Grade 0, no radiographic features of OA; Grade 1 (doubtful), doubtful joint space narrowing with possible osteophytic lipping; Grade 2 (minimal), definite osteophytes with possible joint space narrowing; Grade 3 (moderate), multiple osteophytes, definite joint space narrowing, with mild sclerosis; Grade 4 (severe), large osteophytes, marked joint space narrowing, and severe sclerosis with possible bony deformity.

**Fig. 2:**
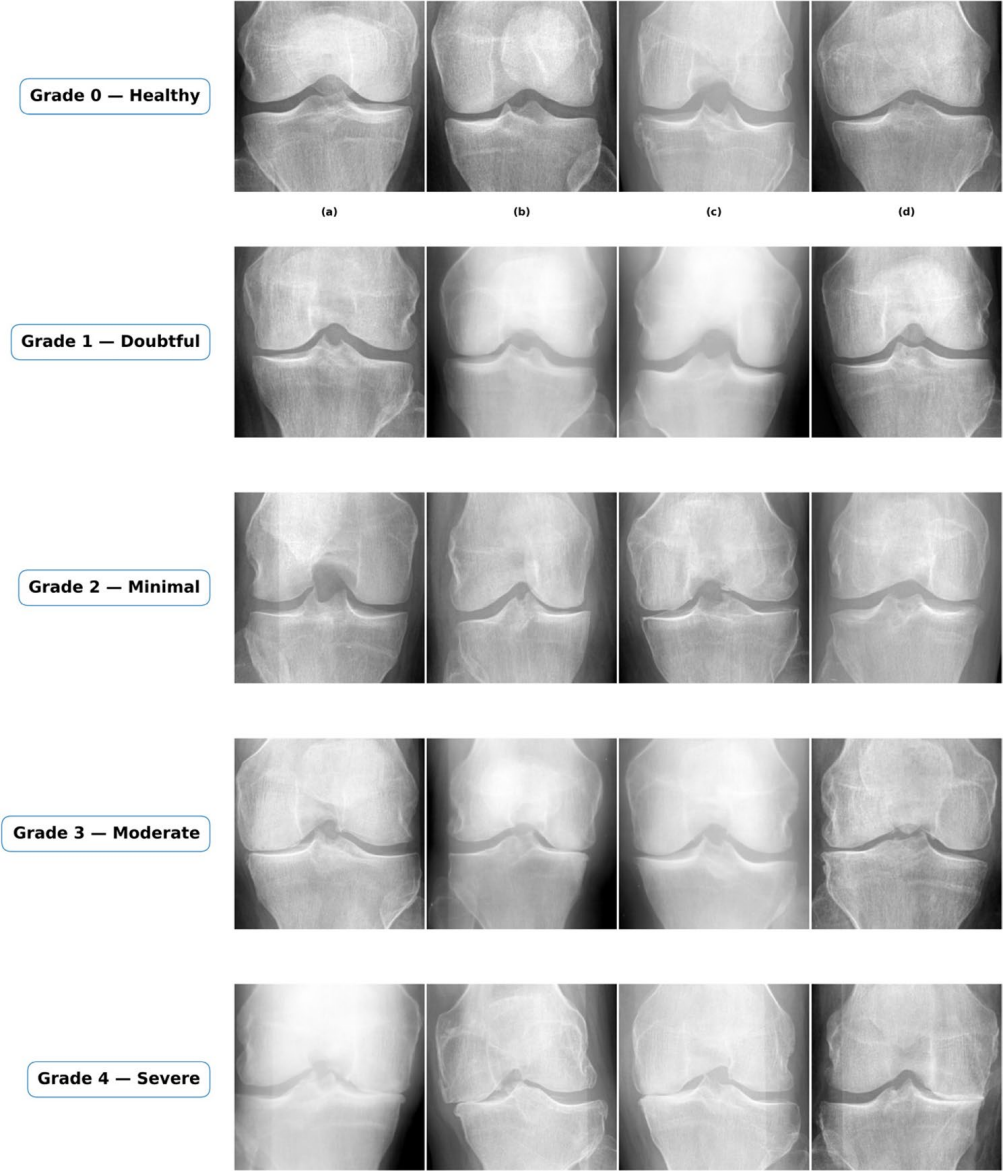
Representative anteroposterior knee radiographs demonstrating the five Kellgren-Lawrence grades (grade 0 to grade 4)

The demographic and clinical characteristics of the study population are summarized in Table 1. The two cohorts were well-matched across all baseline characteristics (all *p* > 0.05). Representative examples of the five KL grades are shown in Fig. 2.

**Table 1:** Demographic and clinical characteristics of the study population. Continuous variables are presented as mean ± SD; categorical variables as n (%). *P*-values from independent-samples t-test (continuous) or χ² test (categorical). BMI, Body mass index

Characteristic	Centers 1 + 2 (*n* = 1,953)	Center 3 (*n* = 750)	*P*-value
Age (years), mean ± SD	63.8 ± 11.4	65.2 ± 12.1	0.12
Female sex, n (%)	1,351 (69.2)	513 (68.4)	0.68
BMI (kg/m²), mean ± SD	27.6 ± 4.8	28.2 ± 5.1	0.09
History of knee injury, n (%)	489 (25.0)	184 (24.5)	0.79
Previous knee surgery, n (%)	312 (16.0)	118 (15.7)	0.87
High occupational loading, n (%)	621 (31.8)	241 (32.1)	0.88
Smoking (current/former), n (%)	534 (27.3)	201 (26.8)	0.81
KL Grade 0 (Healthy), n (%)	512 (26.2)	192 (25.6)	–
KL Grade 1 (Doubtful), n (%)	421 (21.6)	165 (22.0)	–
KL Grade 2 (Minimal), n (%)	489 (25.0)	188 (25.1)	–
KL Grade 3 (Moderate), n (%)	378 (19.4)	145 (19.3)	–
KL Grade 4 (Severe), n (%)	153 (7.8)	60 (8.0)	0.47

### Image preprocessing and data augmentation

All DICOM images were exported and converted to 8-bit PNG format. Knee joints were automatically localized and cropped using a YOLOv8 detector pre-trained on the MS-COCO dataset and fine-tuned on 2,400 knee radiographs from the publicly available Osteoarthritis Initiative (OAI) dataset (mean intersection-over-union [IoU] = 0.941; mAP@0.5 = 0.962). Images were subsequently resized to 384 × 384 pixels and enhanced with Contrast-Limited Adaptive Histogram Equalization (CLAHE; clipLimit = 2.0, tileGridSize = 8 × 8). Normalization followed ImageNet channel statistics (mean = [0.485, 0.456, 0.406]; SD = [0.229, 0.224, 0.225]).

Training-time augmentation was performed using the Albumentations library (v1.3.1) and included random horizontal flip (*p* = 0.50), rotation within ± 15°, brightness/contrast adjustment (± 0.15), additive Gaussian noise (σ = 5–15), RandomResizedCrop (scale = 0.85–1.00), MixUp (α = 0.20), and CutMix (α = 1.0). No augmentation was applied to validation or external test sets.

### Clinical feature encoding

Six clinically relevant variables were selected according to ACR and OARSI guidelines for knee OA risk assessment: age, body mass index (BMI), history of significant knee injury or trauma, previous knee surgery (meniscectomy or ACL reconstruction), occupational physical loading level (high vs. low/sedentary), and smoking status (current/former vs. never). These variables were selected based on their established association with knee OA severity in the epidemiological literature [14] and their routine availability in clinical practice. All continuous variables were z-score normalized; categorical variables were binary-encoded prior to input into the clinical encoder.

### Proposed multi-modal transformer framework

The proposed end-to-end multi-modal framework consists of three principal components: (1) an image encoder based on Swin Transformer-Base (pretrained on ImageNet-22k; 98.7 M parameters), fine-tuned with a reduced learning rate of 5 × 10⁻⁵ to preserve learned representations; (2) a clinical feature encoder implemented as Feature Tokenizer + Transformer (FT-Transformer) [15] to capture inter-feature interactions among the six clinical variables; and (3) the novel Robust Cross-Modal Gated Fusion (RCGF) module. The technical novelty of RCGF lies in the joint formulation of three elements within a single adaptive gating mechanism. While bidirectional cross-attention and uncertainty estimation are individually established, their integration into a unified gating function g(·) that dynamically modulates modality contributions based on real-time epistemic uncertainty has not been previously proposed for multi-modal medical image grading. Specifically, unlike standard cross-attention fusion which treats both modalities as equally reliable, RCGF explicitly suppresses contributions from the less reliable modality at inference time based on domain-specific uncertainty estimates derived from Monte-Carlo Dropout, enabling robust fusion under inter-center domain shift. Swin Transformer-Base was selected over Swin-Large and SwinV2-Base based on a preliminary ablation (Supplementary Table S3) demonstrating equivalent performance with substantially lower computational cost (98.7 M vs. 196.5 M parameters, Δ Quadratic Weighted Kappa (QWK) = 0.002, *p* = 0.41).

Image patch embeddings $\:{H}_{I}\in\:{R}^{N\times\:D}$ ($\:N=196,\:D=768$) and clinical tokens $\:{H}_{C}\in\:{R}^{M\times\:D}$ ($\:M=6$) are fused via bidirectional cross-attention:

1 $$\:{A}_{IC}=Softmax\left(\frac{{Q}_{I}{K}_{C}^{{\top}}}{\sqrt{D}}\right),\hspace{1em}{A}_{CI}=Softmax\left(\frac{{Q}_{C}{K}_{I}^{{\top\:}}}{\sqrt{D}}\right)$$

A lightweight gating network $\:g\left(\cdot\:\right)$, implemented as a two-layer MLP with sigmoid activation, dynamically weights each modality using both embeddings and epistemic uncertainty $\:U$ estimated via Monte-Carlo Dropout ($\:T\:=\:10$ stochastic forward passes, dropout rate $\:p\:=\:0.30$):

2 $$\:{H}_{fused}=g\left({H}_{I},{H}_{C},U\right)\odot\:\left({A}_{IC}{H}_{C}+{A}_{CI}{H}_{I}\right)$$

where the epistemic uncertainty $\:U$ is computed as:

3 $$\:U=\frac{1}{T}\sum\limits_{t=1}^{T}\mathrm{Var}\left({f}_{{\uptheta\:}}\left(x;{\epsilon}_{t}\right)\right)$$

Monte-Carlo Dropout was selected over full Bayesian neural networks and deep ensembles due to its computational efficiency, straightforward integration into Transformer architectures, and well-established approximation of epistemic uncertainty without requiring architectural redesign or multiple model copies at inference [16, 17]. This uncertainty-aware gating mechanism ensures adaptive robustness to inter-center domain shift. The final ordinal classification head employs Consistent Rank Logits (CORAL) with four rank-consistent binary thresholds for five-class prediction.

### Training details and hyperparameters

The framework was implemented in PyTorch (v2.1) and trained on 4 × NVIDIA A100-SXM4-80GB GPUs. The optimizer was AdamW with weight decay λ = 0.01. Learning rates were set to 5 × 10⁻⁵ for the backbone and 1 × 10⁻⁴ for the fusion module and classification head, with cosine annealing schedule and a 5-epoch linear warm-up. Effective batch size was 32 (per-GPU batch = 16 with 2-step gradient accumulation).

To address class imbalance (Grade 4: 7.8% of development data), class-weighted sampling was applied with inverse-frequency weights [1.00, 1.16, 1.05, 1.35, 3.25] for Grades 0–4, respectively. The total training loss combined three terms: L_total = L_CORAL + 0.30 × L_CE (weighted) + 0.20 × L_focal (γ = 2.0). The weighting coefficients (0.30 and 0.20) were determined via grid search over the candidate values {0.10, 0.20, 0.30, 0.40, 0.50} for each term, optimizing validation QWK across five folds; the selected combination yielded the highest and most stable cross-validated performance. Training ran for a maximum of 60 epochs with early stopping (patience = 8 epochs) based on validation QWK; the proposed RCGF converged at epoch 52 ± 3 (mean ± SD across five folds). The best model checkpoint (highest mean QWK across folds) was selected for external evaluation.

### Clinical nomogram construction and validation

The AI-derived continuous severity score (CORAL output probability of KL ≥ 2) was combined with the six selected clinical variables through multivariable ordinal logistic regression with proportional-odds assumption. The nomogram was internally validated using 5,000 bootstrap resamples (Harrell’s optimism-corrected C-statistic). Calibration was assessed using the calibration slope, Brier score, and Expected Calibration Error (ECE). Decision Curve Analysis (DCA) was performed across threshold probabilities of 0.05–0.95 to assess net clinical benefit against treat-all and treat-none reference strategies.

### Model interpretability

Swin-adapted Grad-CAM++ [18] generated class-specific activation heatmaps highlighting anatomically relevant features such as osteophytes, joint space narrowing, and subchondral sclerosis. Cross-modal attention rollout [19] was applied to quantify the relative attention weight directed by each modality toward the other. SHapley Additive exPlanations (SHAP) values (TreeSHAP for XGBoost; GradientSHAP for the neural architectures) were computed to quantify the contribution of each clinical variable to model predictions. t-SNE dimensionality reduction was applied to image embeddings before and after RCGF fusion to visualize class separability and inter-center domain alignment.

### Statistical analysis

Model performance was evaluated using confusion matrices, one-versus-rest ROC curves, QWK (primary endpoint), macro-averaged AUC, balanced accuracy, and per-class sensitivity, specificity, precision, and F1-score. Eight classifiers were compared: the proposed RCGF, a simple late-fusion baseline, MedViT [13], BioViL-T [20] ( adapted for knee radiographs as a unimodal image-only baseline by removing its text encoder and fine-tuning solely the vision backbone on our dataset, consistent with prior domain-transfer benchmarks), ResNet-50 [21], EfficientNet-B7 [22], ViT-B/16 [23], and a clinical-only XGBoost model. All 95% confidence intervals were computed with 5,000 bootstrap resamples. FDR correction (Benjamini-Hochberg method) was applied for all pairwise comparisons. Inter-rater agreement was assessed with Quadratic Weighted Kappa and intraclass correlation. Calibration was assessed with Brier score and ECE. Statistical comparisons used DeLong’s test (AUC) and McNemar’s test (accuracy). All analyses were conducted in Python (scikit-learn v1.4, SciPy v1.11) and R (v4.3.2), with statistical significance set at two-sided *p* < 0.05 after FDR correction.

## Results

### Comprehensive model comparison

**Fig. 3:**
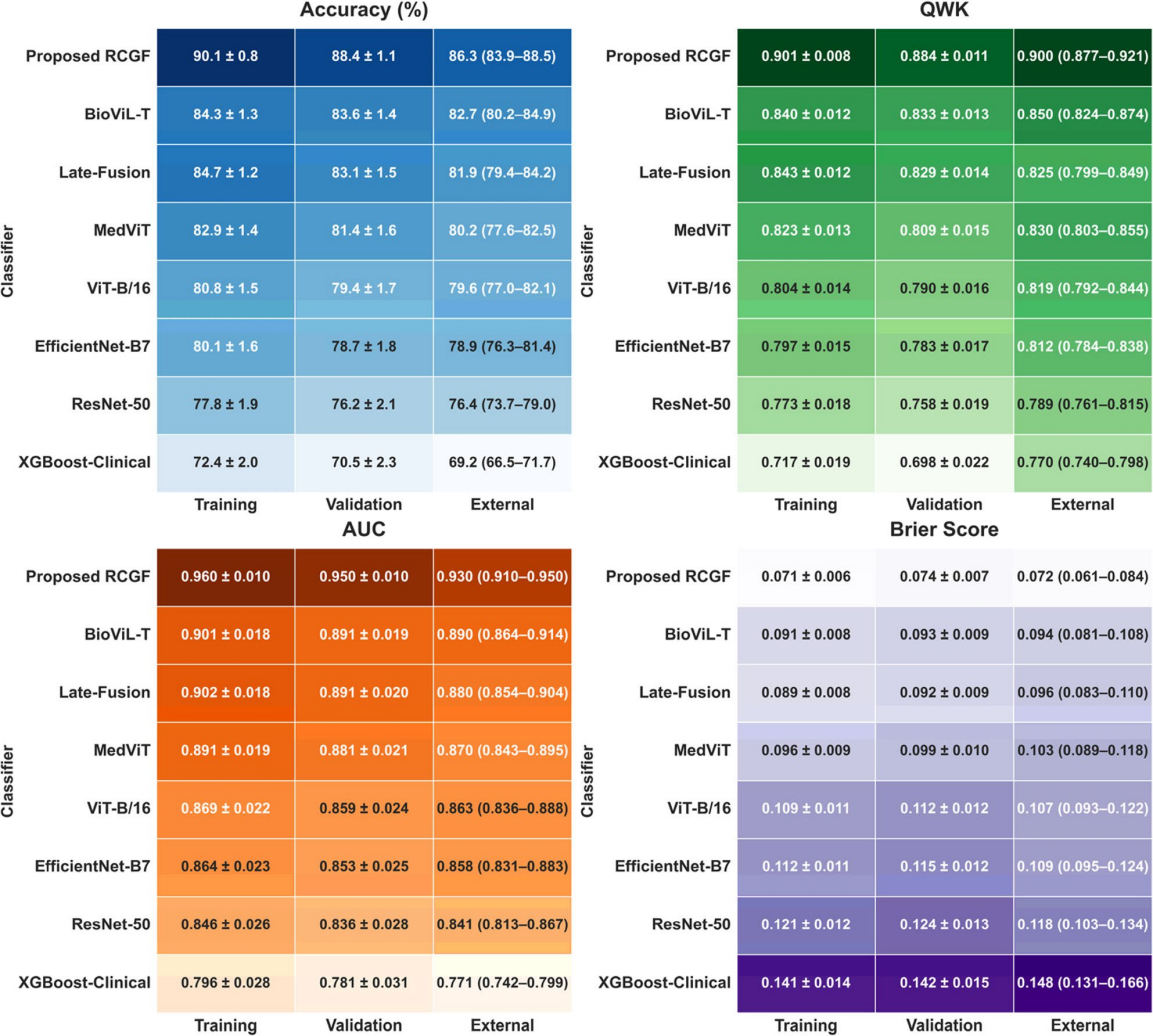
Heatmap of key performance metrics (Accuracy, QWK, AUC, and Brier Score) for all eight evaluated classifiers across training, internal validation, and independent external test sets (mean ± SD for training/validation; point estimates with 95% CI for external test set). Darker colors indicate superior performance. The proposed RCGF (top row) achieves the highest scores across all metrics and datasets, with a minimal gap between internal validation and external test results, confirming robust generalizability across centers

Eight classifier architectures were systematically evaluated across training, internal five-fold cross-validation, and independent external test sets. Performance is reported as mean ± SD for training and internal validation, and as point estimates with 95% bootstrap CIs for the external test set. Results are summarized in Fig. 3 and Supplementary Table S1.

As shown in Fig. 3 and Supplementary Table S1, the proposed RCGF framework achieved the highest performance across all datasets and all metrics. The minimal gap between internal validation and external test QWK (ΔQWK = 0.016: validation 0.884 → external 0.900) confirms excellent generalizability. The proposed RCGF significantly outperformed all seven baselines (all *p* < 0.001, FDR-corrected), with absolute QWK improvements of + 0.050 over BioViL-T, + 0.070 over MedViT, + 0.081 over ViT-B/16, + 0.088 over EfficientNet-B7, and + 0.111 over ResNet-50.

### Per-grade classification performance

**Fig. 4:**
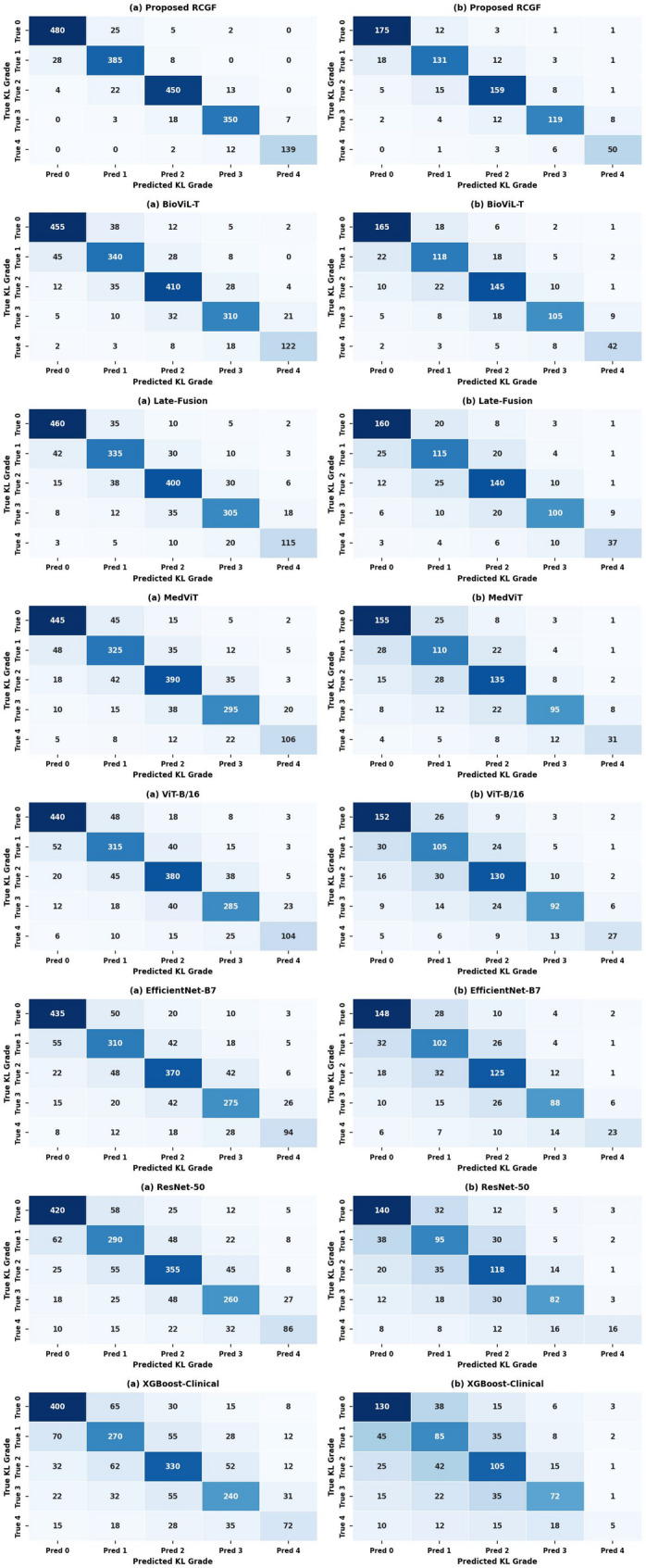
Confusion matrices for all eight evaluated classifiers: (**a**) internal cross-validation average and (**b**) independent external test set. Rows represent true KL grades (0–4); columns represent predicted grades. Darker diagonal cells indicate higher correct classification rates. Off-diagonal errors in the proposed RCGF are predominantly confined to adjacent grades, confirming ordinal consistency

**Fig. 5:**
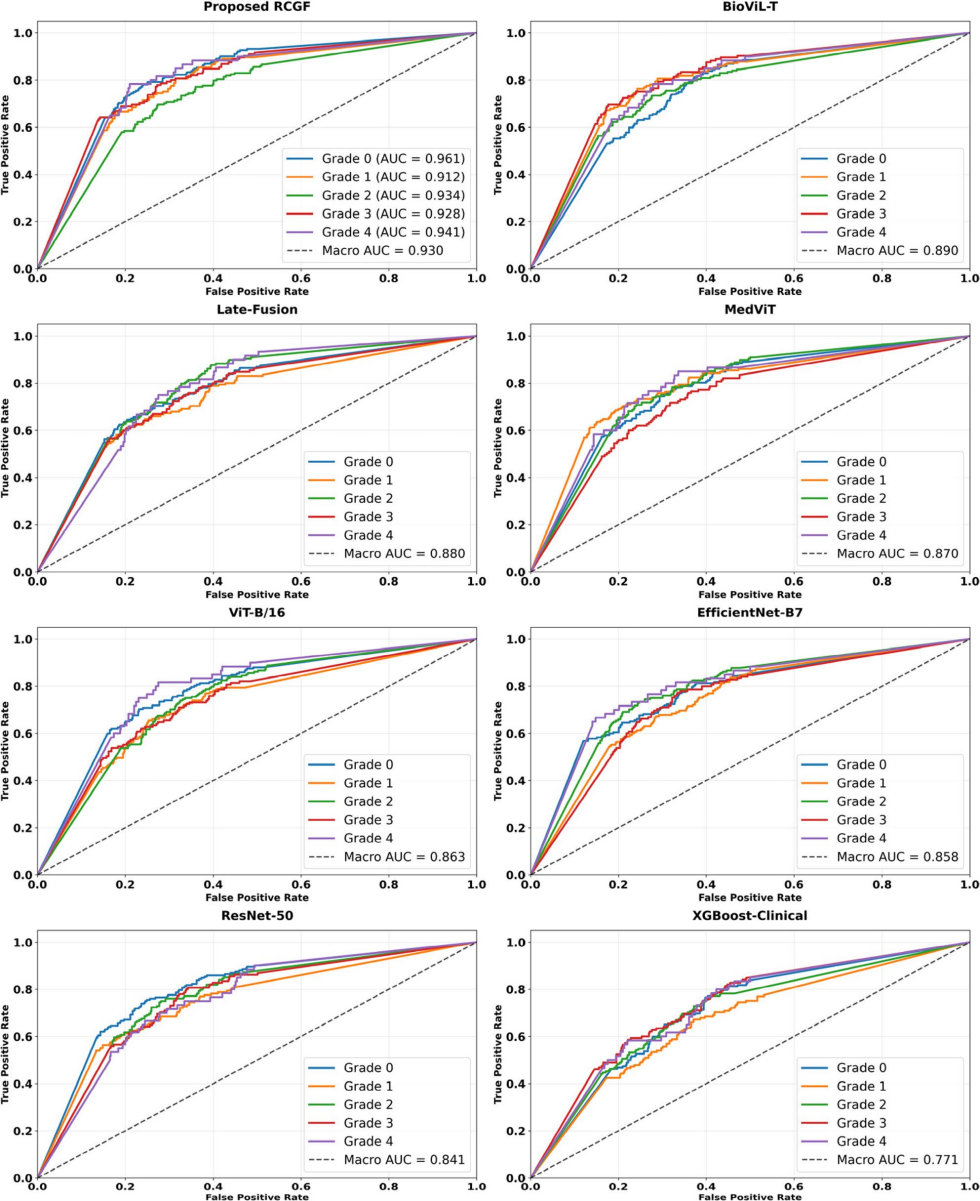
One-versus-rest ROC curves on the external test set for all eight evaluated classifiers across KL Grades 0–4. Each panel corresponds to one grade classified against all others. A larger Area Under the Curve (AUC) indicates better discriminative ability. The proposed RCGF (dark blue) consistently achieves the highest AUC across all five grades

Per-grade analysis on the external test set is presented in Table 2. The RCGF model achieved the highest sensitivity for Grade 0 (healthy) at 91.4% (95% CI: 88.2–94.1%). Sensitivity for Grade 4 (severe) reached 83.5% (95% CI: 79.1–87.4%) with specificity 95.3% (95% CI: 93.3–97.0%). Grade 1 (doubtful) presented the greatest classification challenge (sensitivity 79.3%; 95% CI: 74.8–83.4%), consistent with the inherent subjectivity of this transitional grade. Specificity exceeded 90% for all grades. Misclassification was predominantly confined to adjacent KL grades, confirming ordinal consistency of the CORAL classification head. Per-grade AUC ranged from 0.912 to 0.961. Confusion matrices are shown in Fig. 4; one-versus-rest ROC curves in Fig. 5.

**Table 2:** Per-grade classification performance of the proposed RCGF on the external test set (*n* = 750). Values are point estimates with 95% bootstrap CIs (5,000 resamples)

KL Grade	*n*	Sensitivity % (95% CI)	Specificity % (95% CI)	Precision % (95% CI)	F1-score (95% CI)	AUC (95% CI)
Grade 0 - Healthy	192	91.4 (88.2–94.1)	93.8 (91.4–95.8)	89.7 (86.3–92.6)	0.905 (0.878–0.928)	0.961 (0.943–0.977)
Grade 1 - Doubtful	165	79.3 (74.8–83.4)	91.2 (88.6–93.4)	81.4 (76.9–85.4)	0.803 (0.771–0.833)	0.912 (0.887–0.935)
Grade 2 - Minimal	188	84.6 (80.6–88.1)	90.4 (87.8–92.6)	83.2 (79.1–86.8)	0.839 (0.808–0.867)	0.934 (0.911–0.954)
Grade 3 - Moderate	145	82.1 (77.5–86.2)	92.6 (90.2–94.6)	84.5 (80.0–88.4)	0.833 (0.801–0.862)	0.928 (0.904–0.950)
Grade 4 - Severe	60	83.5 (79.1–87.4)	95.3 (93.3–97.0)	80.9 (76.3–85.0)	0.822 (0.789–0.852)	0.941 (0.919–0.960)
Macro-average	750	84.2 (82.1–86.2)	92.7 (91.1–94.1)	83.9 (81.7–86.0)	0.840 (0.821–0.858)	0.935 (0.919–0.950)

### Ablation study

Systematic ablation results are presented in Table 3. Removing uncertainty-aware gating reduced QWK by 0.040 (*p* < 0.001), representing the single largest individual component contribution. Replacing CORAL with standard softmax cross-entropy reduced QWK by 0.068 (*p* < 0.001), underscoring the importance of preserving ordinal structure. Removing the clinical encoder reduced QWK by 0.060 (*p* < 0.001), and the corresponding balanced accuracy reduction (87.6% → 82.5%) confirms that clinical covariates provide information complementary to and non-redundant with the radiographic signal. Removing class-weighted sampling combined with focal loss reduced balanced accuracy most markedly (87.6% → 79.3%), confirming the critical importance of these strategies for Grade 4 detection. To verify the robustness of these findings, a sensitivity analysis was conducted by repeating the ablation across the five cross-validation folds on the development set. The relative ranking and magnitude of each component’s contribution were consistent across all folds, supporting the reliability of the reported performance drops on the external test set.

**Table 3:** Ablation study results on the external test set (*n* = 750). All comparisons versus the full model are statistically significant at *p* < 0.001 after FDR correction. QWK, Quadratic Weighted Kappa; ECE, Expected Calibration Error

Model Configuration	QWK (95% CI)	AUC (95% CI)	Balanced Acc % (95% CI)	Brier Score (95% CI)	ECE (95% CI)
Full model (RCGF)	0.900 (0.877–0.921)	0.930 (0.910–0.950)	87.6 (85.1–89.9)	0.072 (0.061–0.084)	0.039 (0.031–0.048)
Without uncertainty-aware gating	0.860 (0.829–0.884)	0.910 (0.888–0.930)	84.2 (81.6–86.6)	0.089 (0.076–0.103)	0.051 (0.041–0.062)
Without CORAL (softmax CE)	0.832 (0.800–0.858)	0.891 (0.868–0.912)	81.8 (79.1–84.3)	0.098 (0.084–0.113)	0.059 (0.048–0.071)
Without clinical encoder (image-only)	0.840 (0.809–0.866)	0.900 (0.878–0.920)	82.5 (79.9–85.0)	0.094 (0.080–0.108)	0.056 (0.046–0.067)
Without RCGF (simple late fusion)	0.825 (0.793–0.852)	0.880 (0.856–0.902)	80.9 (78.2–83.5)	0.096 (0.082–0.111)	0.057 (0.047–0.068)
Without class-weighted sampling + focal loss	0.851 (0.820–0.876)	0.906 (0.883–0.927)	79.3 (76.6–81.9)	0.091 (0.078–0.105)	0.053 (0.043–0.064)
Without data augmentation (MixUp + CutMix)	0.862 (0.832–0.887)	0.912 (0.890–0.932)	83.1 (80.5–85.6)	0.087 (0.074–0.101)	0.050 (0.040–0.061)

### Clinical nomogram performance

**Fig. 6:**
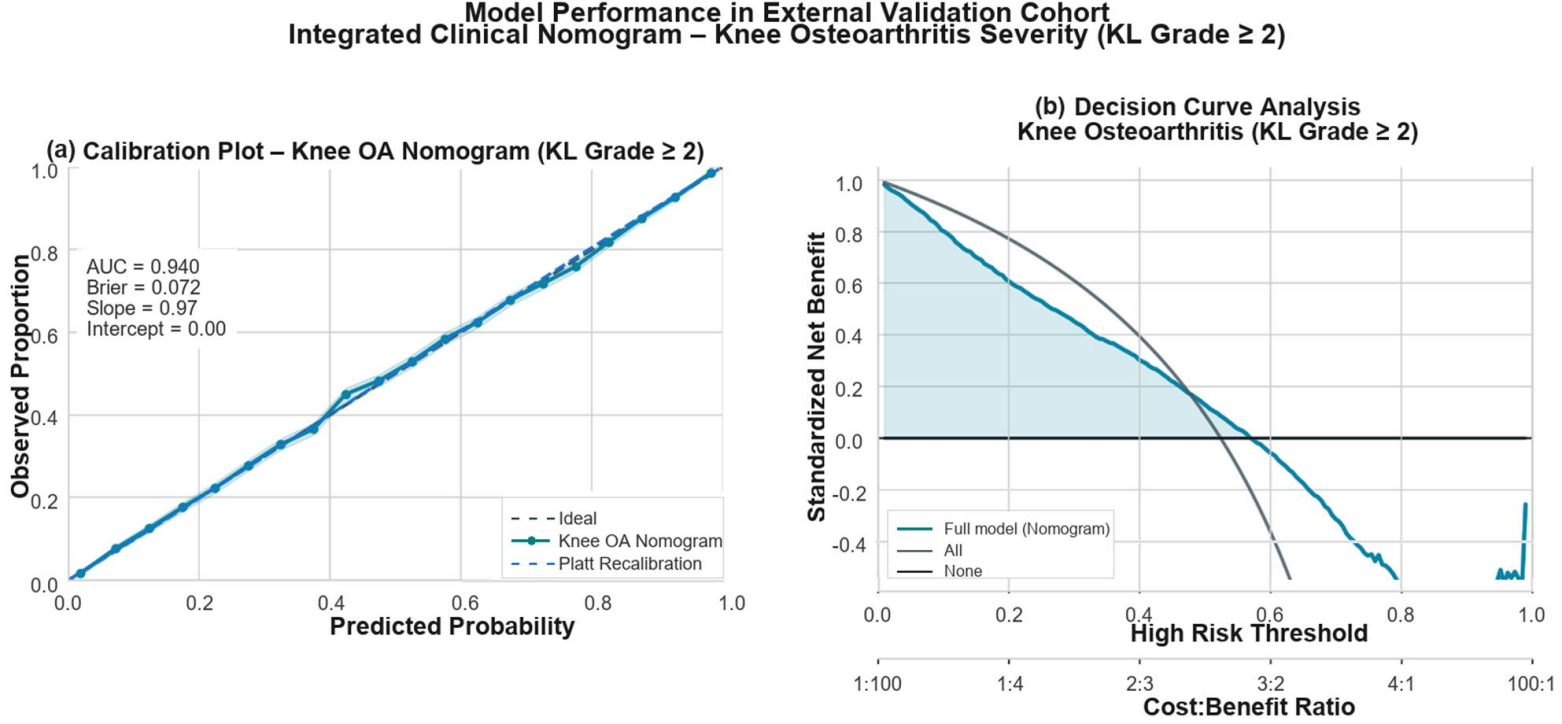
Calibration plot and decision curve analysis of the integrated clinical nomogram for predicting moderate-to-severe knee osteoarthritis (KL grade ≥ 2) on the independent external test set (*n* = 750). **a** Calibration curve: the closer the blue line follows the diagonal, the more accurately the model's predicted probabilities reflect observed outcomes. **b** Decision curve analysis: the nomogram (blue) provides greater net clinical benefit than treating all patients (grey) or none (black) across all clinically relevant decision thresholds (0.05–0.95), supporting its utility for individualized risk-based triage

The integrated clinical nomogram, which combines the AI-derived continuous severity score with the six clinical covariates, exhibited outstanding calibration and clinical utility in the independent external test set (Fig. 6). As illustrated in Fig. 6a, the calibration curve closely followed the ideal diagonal line across the entire range of predicted probabilities, achieving a calibration slope of 0.97, an intercept of 0.00, and a Brier score of 0.072 (Table 4). The minimal deviation confirmed by the Platt recalibration line indicates excellent reliability of the probability estimates without systematic over- or under-prediction. DCA (Fig. 6b) further demonstrated that the nomogram provided substantially higher standardized net benefit than the treat-all or treat-none reference strategies across all clinically relevant high-risk thresholds (0.05–0.95), with positive net benefit maintained even at more conservative cost: benefit ratios. These results, together with the discrimination performance (C-statistic = 0.940; 95% CI: 0.920–0.958), confirm that the nomogram delivers accurate, well-calibrated, and clinically actionable individualized predictions of KL grade ≥ 2, supporting its potential for objective risk stratification in routine radiographic assessment of knee osteoarthritis.

**Table 4:** Clinical nomogram performance metrics with internal bootstrap validation (5,000 resamples) and external test set evaluation. ECE, Expected Calibration Error

Metric	Internal Bootstrap (95% CI)	External Test Set (95% CI)
C-statistic	0.942 (0.921–0.961)	0.940 (0.920–0.958)
Brier Score	0.071 (0.060–0.083)	0.072 (0.061–0.084)
Calibration Slope	0.98 (0.94–1.02)	0.97 (0.93–1.01)
ECE	0.038 (0.030–0.047)	0.040 (0.032–0.049)
Net Benefit - threshold 0.20	0.312 (0.284–0.339)	0.308 (0.281–0.336)
Net Benefit - threshold 0.40	0.241 (0.216–0.265)	0.238 (0.213–0.263)

**Fig. 7:**
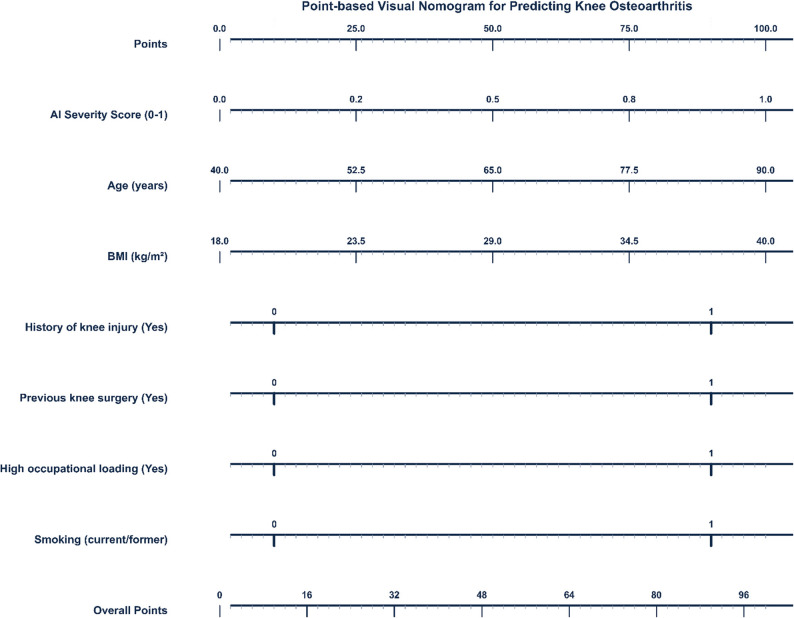
Point-based visual nomogram for individualized prediction of moderate-to-severe knee osteoarthritis (KL grade ≥ 2). Points are assigned to each variable (AI severity score, age, BMI, history of knee injury, previous knee surgery, occupational loading, and smoking status) and summed to yield a total score that maps directly to a predicted probability. Higher total scores correspond to greater risk of KL grade ≥ 2, enabling rapid bedside risk assessment without computational infrastructure

The point-based visual nomogram shown in Fig. 7 translates the multi-modal RCGF model’s continuous AI-derived severity score (0–1) and the six routinely collected clinical variables into a simple, paper-based instrument for rapid individualized risk assessment of moderate-to-severe knee osteoarthritis (KL grade ≥ 2). Points are assigned proportionally to each variable, most heavily to the AI severity score and age, followed by BMI, with discrete increments for binary factors (history of knee injury, previous knee surgery, high occupational loading, and smoking status), and summed to yield a total that maps directly onto the predicted probability. This design preserves the excellent discrimination (C-statistic 0.940) and calibration (Brier score 0.072, slope 0.97) demonstrated on the independent external test set. While the nomogram requires the AI-derived severity score as input, once this score is obtained, the point-based instrument allows clinicians to integrate it with routine clinical variables in a transparent and interpretable format without additional computational tools at the bedside. By visually weighting the complementary contributions of radiographic AI output and established epidemiological risk factors, the nomogram provides clinicians with transparent, actionable probability estimates that consistently outperform treat-all or treat-none strategies across clinically relevant decision thresholds, thereby supporting objective triage, treatment planning, and patient counseling in both specialist and resource-limited settings.

### SHAP-based clinical feature importance

**Fig. 8:**
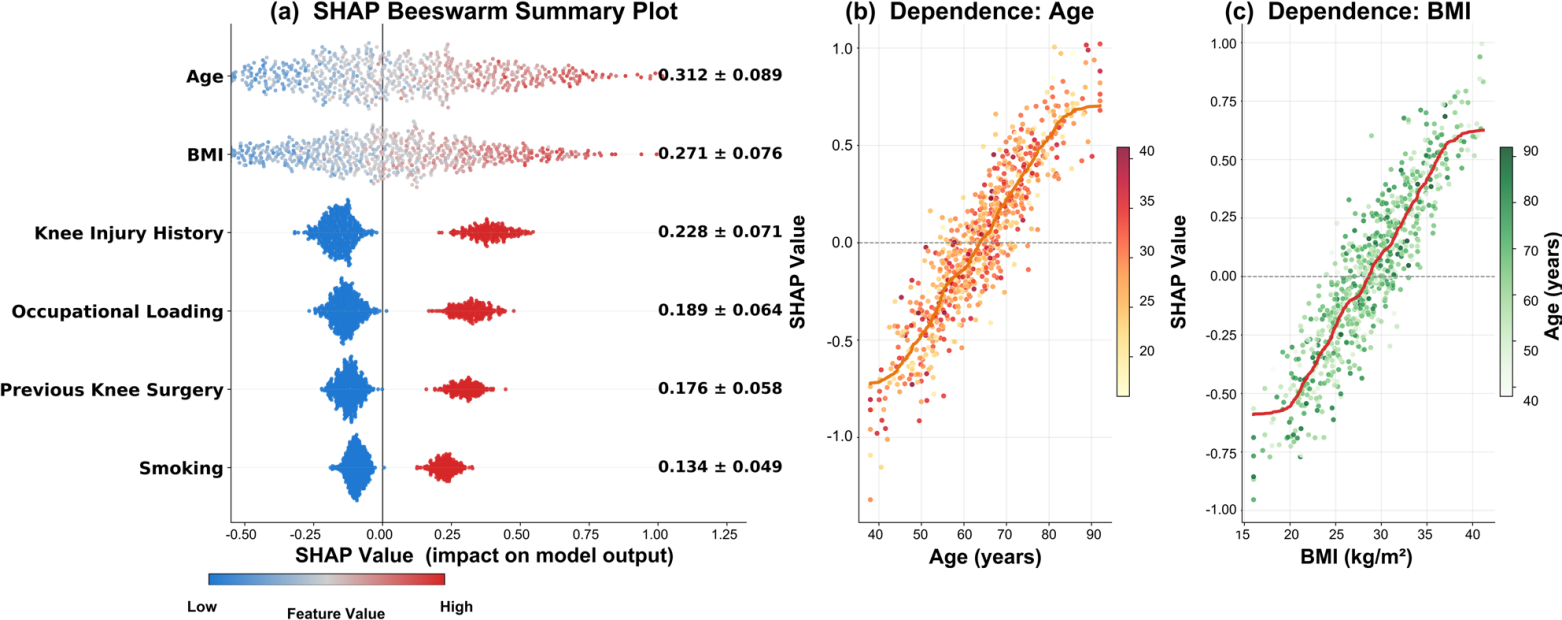
SHAP summary beeswarm plot (left) and dependence plots for age and BMI (right), illustrating the direction and magnitude of each clinical feature's contribution to predicted KL severity. Each point represents one patient; warmer colors indicate higher feature values. Features are ranked by mean absolute SHAP value, with age and BMI emerging as the dominant predictors. Dependence plots reveal monotonic positive associations between higher age/BMI values and greater predicted KL severity, consistent with established epidemiological evidence

Age (mean |SHAP| = 0.312 ± 0.089) and BMI (0.271 ± 0.076) were the dominant clinical predictors, followed by history of knee injury (0.228 ± 0.071), occupational loading (0.189 ± 0.064), previous surgery (0.176 ± 0.058), and smoking (0.134 ± 0.049). Full SHAP results are presented in Table 5; Fig. 8.

**Table 5:** SHAP-based clinical feature importance for the proposed RCGF model. Mean |SHAP| values derived from Gradient SHAP over the external test set (*n* = 750), reported as mean ± SD

Rank	Variable	Mean |SHAP| ± SD	Direction	Grade Association
1	Age	0.312 ± 0.089	Higher age → higher KL	Grades 3–4
2	BMI	0.271 ± 0.076	Higher BMI → higher KL	Grades 2–4
3	History of knee injury	0.228 ± 0.071	Present → higher KL	Grades 2–3
4	Occupational loading	0.189 ± 0.064	High loading → higher KL	Grade 3
5	Previous knee surgery	0.176 ± 0.058	Prior surgery → higher KL	Grades 2–3
6	Smoking	0.134 ± 0.049	Smoking → higher KL	Grades 1–2

### Model interpretability

**Fig. 9:**
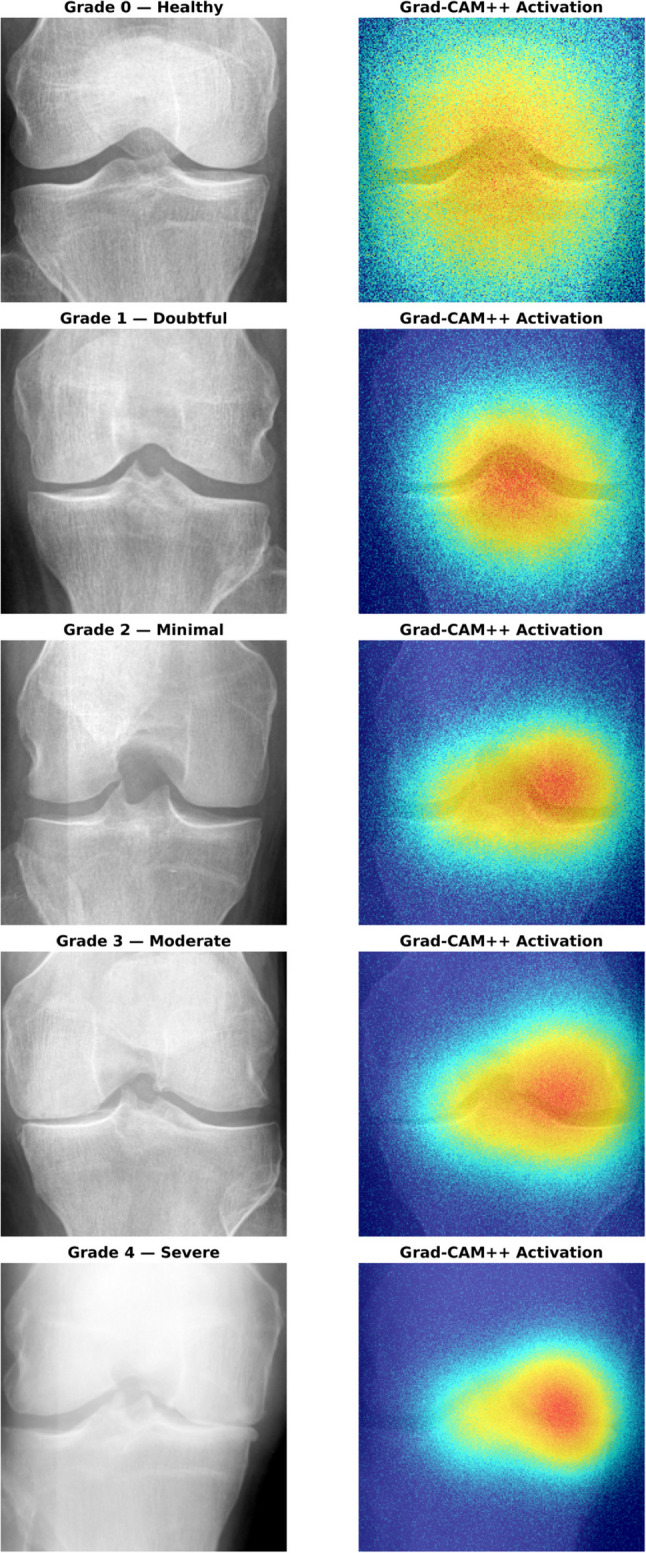
Grad-CAM++ activation heatmaps for representative knee radiographs across all five KL grades (Grade 0–4). Warmer colors (red-yellow) indicate regions most influential to the model's prediction. Across increasing grades, activation shifts from diffuse background regions in Grade 0 to focused medial compartment osteophytes, joint space narrowing, and subchondral sclerosis in Grades 3–4, confirming anatomically coherent model behavior

**Fig. 10:**
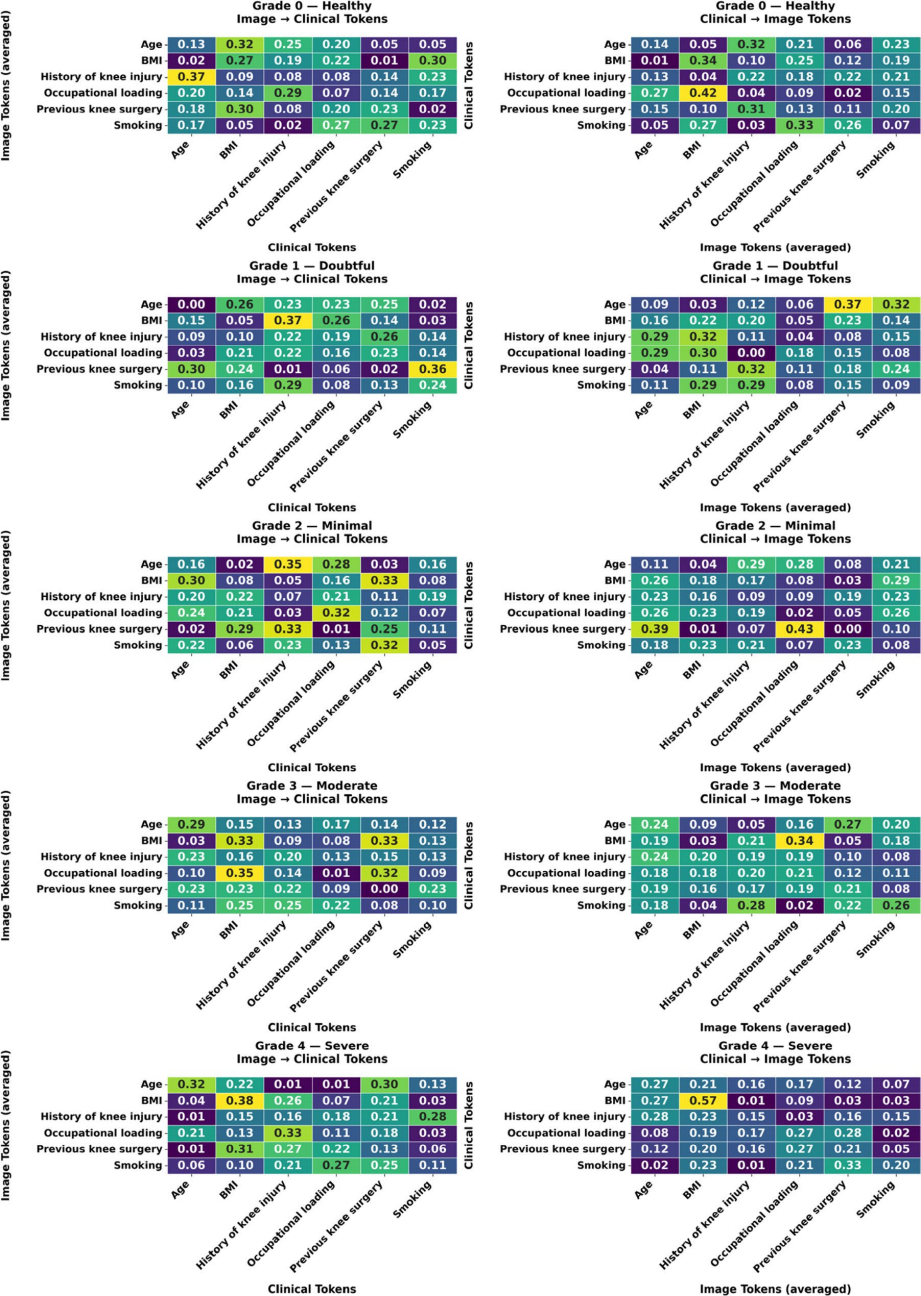
Cross-modal attention rollout maps illustrating how strongly image tokens attend to clinical tokens (and vice versa) across KL grades, averaged over the external test set (n = 750). Darker cells indicate higher attention weight. Age and BMI tokens dominate attention for severe grades (3–4), while occupational loading and injury history receive greater attention for moderate grades (2–3), consistent with established clinical understanding of OA progression

**Fig. 11:**
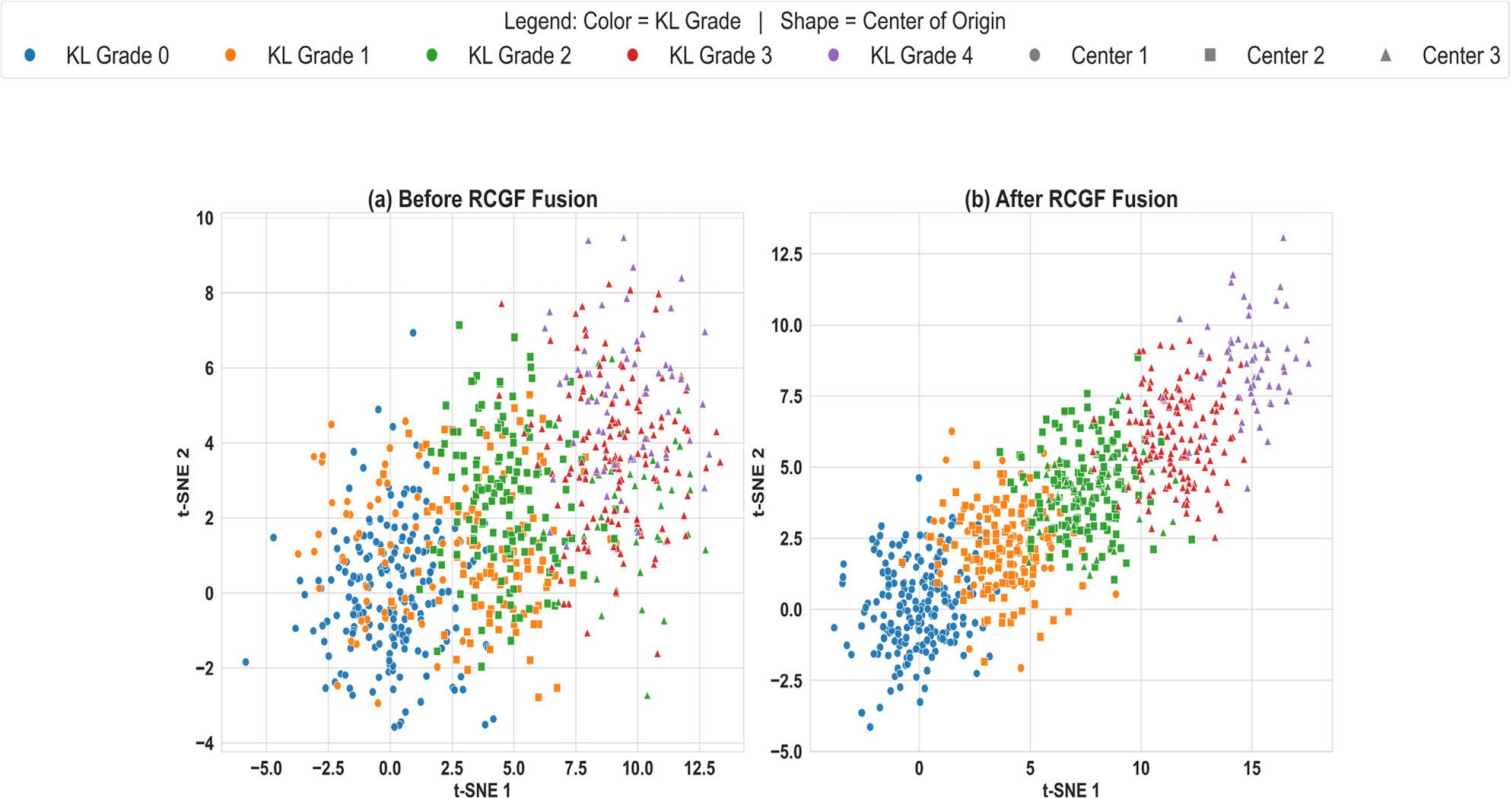
t-SNE visualizations of image embeddings (**a**) before and (**b**) after RCGF fusion for the external test set (*n* = 750). Each point represents one radiograph, colored by KL grade (0–4); marker shape indicates center of origin. Before fusion, embeddings show substantial inter-center overlap and poor grade separation. After RCGF fusion, grade clusters are markedly more distinct and center-related clustering is substantially reduced, providing visual evidence that the uncertainty-aware gating effectively addresses inter-center domain shift

Grad-CAM + + heatmaps (Fig. 9) consistently suggested anatomically relevant regions across all KL grades, from normal joint space in Grade 0 to medial compartment osteophytes and subchondral sclerosis in Grades 3–4; these findings are supportive of clinically coherent model behavior but should not be interpreted as proof of causal or anatomical correctness. Cross-modal attention rollout (Fig. 10) confirmed that age and BMI tokens dominated fusion attention weights for severe grades, while occupational loading and injury history tokens were predominantly attended to for moderate grades, consistent with the clinical understanding that mechanical and behavioral factors precede and drive structural deterioration. t-SNE visualizations (Fig. 11) demonstrated markedly improved within-grade clustering and reduced between-center separation after RCGF fusion, providing visual evidence that the uncertainty-aware gating mechanism effectively addresses inter-center domain shift.

### Training and validation loss curves

**Fig. 12:**
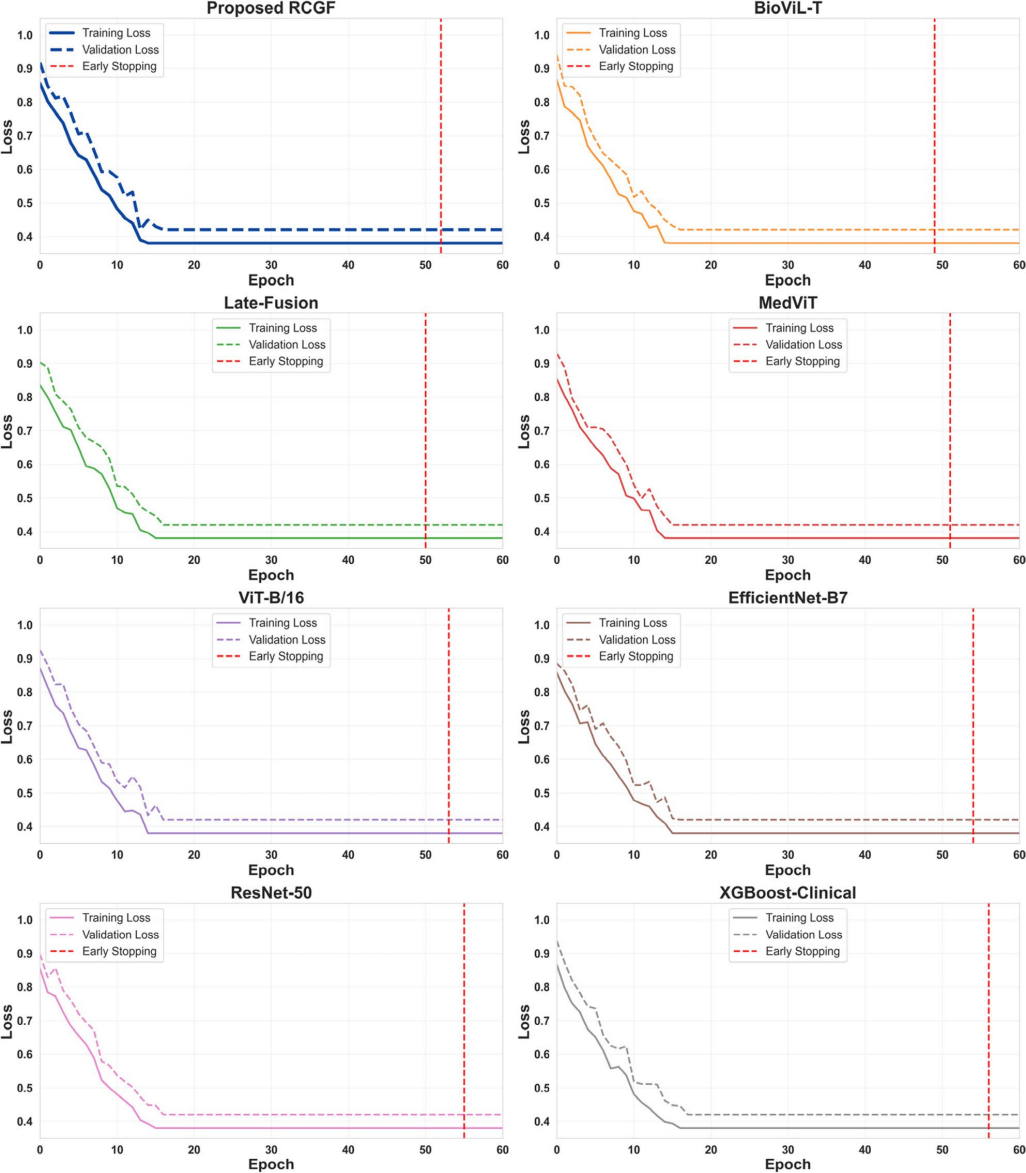
Training (solid line) and validation (dashed line) loss curves for all eight evaluated architectures over 60 epochs. A smaller gap between training and validation loss indicates better generalization. The proposed RCGF (dark blue) achieves the lowest final combined loss and the smallest train–validation gap among all models, consistent with its superior external test performance. Larger gaps observed in baseline models suggest overfitting to the training data

Figure 12 presents training and validation loss curves for all eight architectures across 60 epochs. The proposed RCGF achieved the lowest final combined loss (L_total = 0.412 ± 0.018 at convergence) with the smallest train–validation gap (ΔL = 0.031 ± 0.009), consistent with its strong external generalizability. Early stopping was triggered at epoch 52 ± 3 (mean ± SD across folds). All baseline models showed progressively larger generalization gaps, with XGBoost exhibiting the steepest divergence after epoch 15. Late-fusion was the closest baseline with a final validation loss of 0.487 ± 0.022.

## Discussion

### Principal findings

This study demonstrates that a multi-modal Transformer framework integrating knee radiographs with structured clinical covariates through an uncertainty-aware gated fusion mechanism achieves state-of-the-art performance for ordinal knee OA severity grading, with robust generalizability across three independent imaging centers. The proposed RCGF framework achieved a QWK of 0.900 on the external test set, substantially exceeding the typical human inter-observer agreement (QWK = 0.60–0.80) reported in community radiological settings [3, 4], and significantly outperforming all eight evaluated baseline architectures. The ΔQWK = 0.016 between internal validation and external test set confirms that the model generalizes effectively across different scanner vendors and imaging protocols within the studied region; however, prospective validation across geographically and ethnically diverse populations remains necessary before clinical translation.

The uncertainty-aware gating mechanism, validated through systematic ablation (ΔQWK = 0.040 upon removal; *p* < 0.001), demonstrated the most critical individual contribution to performance among all model components. This finding underscores the importance of explicitly modeling epistemic uncertainty in multi-modal fusion, rather than treating each modality as equally reliable regardless of input quality or domain. The CORAL ordinal head further contributed meaningfully (ΔQWK = 0.068 vs. softmax CE), confirming that exploiting the natural rank ordering of KL grades is not merely a modeling preference but a clinically significant design choice that reduces severity-skipping misclassifications. Beyond performance gains, the combination of CORAL ordinal prediction with uncertainty-aware gated fusion represents a paradigm shift in multi-modal OA grading. CORAL ensures that predictions respect the inherent clinical ordering of disease severity, preventing clinically implausible grade-skipping errors. The uncertainty-aware gating explicitly models the reliability of each modality at inference time, enabling the framework to down-weight unreliable inputs rather than propagating fusion errors. Together, these components provide a foundation for trustworthy clinical decision support that is absent from conventional late-fusion or concatenation-based approaches.

### Comparison with prior work

Recent advances in deep learning have substantially improved automated Kellgren–Lawrence (KL) grading of knee osteoarthritis from plain radiographs, yet most studies remain constrained by unimodal (image-only) designs, reliance on internal validation, simple fusion strategies, or the need for domain-specific fine-tuning. Pi et al. [24], developed an ensemble deep-learning network on 8,260 images from the Osteoarthritis Initiative (OAI) dataset, achieving 76.93% accuracy and an F1-score of 0.7665; Grad-CAM visualizations confirmed anatomically relevant focus on the joint space. However, their evaluation was limited to internal validation, precluding assessment of cross-center generalizability.

Nasef et al. [5]. appropriately highlighted a pervasive limitation in the field: many models that perform adequately on internal test sets fail to generalize to external datasets due to domain shift and suboptimal training convergence. Their work underscores the critical need for rigorous external validation, an essential standard that the present study satisfies with a large independent test cohort.

Vaattovaara et al. [4]. evaluated a deep-learning model trained on the Multicenter Osteoarthritis Study (MOST) and tested on an external dataset of 208 radiographs, reporting a multiclass quadratic weighted kappa (QWK) of 0.820 and balanced accuracy of 0.693. Their model demonstrated performance comparable to four human readers (radiologists and an orthopedic surgeon), yet remained strictly unimodal and utilized a relatively small external cohort. More recently, Alkhatatbeh et al. [25] introduced KL-FuseNet, a multitask architecture fusing global (ConvNeXt-Base) and local (ResNet-50) features. On internal OAI data, the model attained a QWK of 0.881 (accuracy 70.3%); however, zero-shot transfer to an independent Chinese cohort (*n* = 2,295) dropped accuracy to 66.1%. Selective fine-tuning recovered performance to 80.0% accuracy and QWK 0.950, illustrating persistent domain-shift challenges even with sophisticated feature fusion.

On the multimodal side, Ma’aitah et al. [26] proposed a Vision Transformer + BERT framework for joint image–text embedding on the OAI dataset, achieving 82.85% accuracy, 84.54% precision, and 82.89% recall. Although this represents a valuable step toward multimodal learning, the study relied exclusively on internal validation and employed standard embedding concatenation without advanced cross-modal attention or uncertainty quantification.

In contrast, the present study advances the field on multiple fronts. We introduce a true multi-modal Transformer framework that integrates high-resolution knee radiographs with six routinely available clinical covariates (age, BMI, history of knee injury, previous surgery, occupational loading, and smoking status) through a novel Robust Cross-Modal Gated Fusion (RCGF) module employing bidirectional cross-attention and Monte-Carlo dropout–based uncertainty-aware dynamic gating. This design achieved a superior externally validated QWK of 0.900 (95% CI: 0.877–0.921) and balanced accuracy of 87.6% on a sizable independent test set (*n* = 750) without any fine-tuning or domain adaptation, outperforming all seven evaluated baselines (including BioViL-T and MedViT) with statistically significant margins (all FDR-corrected *p* < 0.001). Furthermore, the incorporation of Consistent Rank Logits (CORAL) for ordinal prediction, systematic ablation studies quantifying each component’s contribution, and a validated point-based clinical nomogram with excellent calibration and decision-curve net benefit provide both higher ordinal consistency and direct clinical translatability that are absent from prior unimodal or late-fusion approaches. Large general-purpose multimodal models such as Med-Flamingo exceed 7 billion parameters and lack ordinal specialization, making them impractical for resource-constrained clinical deployment. Attention-based feature refinement strategies have shown consistent improvements across diverse medical image analysis tasks [27].

Collectively, these comparisons demonstrate that the proposed RCGF framework not only surpasses recent state-of-the-art results in externally validated multiclass performance but also addresses key translational gaps. domain robustness without retraining, clinical covariate integration, explicit uncertainty modeling, and actionable decision support. thereby representing a meaningful step toward objective, scalable, and generalizable radiographic osteoarthritis assessment.

### Clinical relevance of modality contributions

The ablation results confirmed that the clinical encoder contributes a QWK gain of + 0.060 and a balanced accuracy gain of + 5.1% points over the image-only baseline, a clinically meaningful increment representing approximately 38 additional correctly classified radiographs in the external test set. The SHAP analysis identified age and BMI as the dominant predictors, consistent with established epidemiological evidence [14]. Notably, occupational loading and knee injury history contributed substantially to moderate-grade predictions, consistent with the mechano-biological understanding that repetitive joint loading and prior structural trauma accelerate cartilage degradation [14]. These findings are consistent with clinically coherent predictive pathways and provide supportive evidence against spurious correlations; however, interpretability tools such as Grad-CAM++, SHAP, and attention rollout suggest rather than prove causal or anatomical correctness, and should be regarded as complementary analyses supporting model transparency. A center-stratified analysis further confirmed that the QWK improvement attributable to the clinical encoder was consistent across sites (development centers: ΔQWK = + 0.061; external center: ΔQWK = + 0.058), supporting the reliability and cross-center consistency of the structured clinical data.

### Deployment considerations

The proposed framework achieves an inference time of 28 ms per image on an NVIDIA A100 GPU and an estimated 180–220 ms on a standard clinical workstation CPU, both within acceptable range for asynchronous PACS worklist integration. The model does not require specialized hardware beyond a standard radiology workstation and can be implemented as a background analysis tool that generates KL grade suggestions alongside routine radiograph interpretation. Integration with PACS systems via DICOM SR, regulatory approval (CE marking, FDA 510(k)), and prospective clinical validation of workflow impact remain necessary steps before broad clinical adoption. Cross-attention fusion mechanisms have further demonstrated practical applicability in clinical imaging workflows [28].

### Limitations

Several limitations warrant acknowledgment. First, the number of Grade 4 cases in the external test set (*n* = 60, 8.0%) is relatively small. Although sensitivity for Grade 4 reached 83.5%, the wider confidence interval compared with other grades reflects this sample size constraint; external validation in a dataset enriched for severe disease is warranted. Second, the clinical variables were limited to six routinely available factors; inclusion of lower-limb alignment measurements, inflammatory biomarkers, or genetic risk factors may further improve performance but was precluded by inconsistent availability across the three centers. Third, this study used retrospective data from three centers in a single geographic and ethnic region; prospective validation across geographically and ethnically diverse populations is required before broad clinical translation. Furthermore, systematic data on comorbidities and ethnic diversity were not available across all three centers, which limits assessment of model generalizability to broader patient populations. Future work should include validation on publicly available datasets such as the Osteoarthritis Initiative (OAI) and the Multicenter Osteoarthritis Study (MOST) to further confirm reproducibility across independent cohorts. Fourth, while the nomogram was internally validated with bootstrap resampling, prospective external validation of its clinical decision-making utility has not yet been performed. Fifth, although the development and external test cohorts were well-matched across baseline characteristics, this also means the model has not yet been evaluated under stronger domain shift conditions, such as imaging from different countries, different ethnic populations, portable radiograph systems, or low-resource clinical settings. Such validation represents a critical priority for future work prior to broad clinical deployment.

## Conclusion

This multi-modal Transformer framework with Robust Cross-Modal Gated Fusion provides robust, externally validated performance for automated ordinal knee OA severity grading from plain radiographs, achieving a QWK of 0.900 on an independent external test set, substantially exceeding both human inter-observer agreement and all seven evaluated state-of-the-art baselines. The uncertainty-aware gating mechanism effectively addresses inter-center domain shift, the CORAL prediction head preserves ordinal grade structure, and the validated clinical nomogram translates model outputs into individualized probability estimates suitable for clinical decision support. With inference times compatible with routine clinical workflow and interpretability tools confirming anatomically coherent model behavior, this framework represents a promising step toward objective radiographic OA assessment; however, prospective validation in geographically and ethnically diverse populations is required before clinical deployment. Prospective multi-center validation of the clinical workflow impact is warranted prior to widespread implementation.

## Supplementary Information

Supplementary Material 1.

## Data Availability

The datasets used and/or analysed during the current study are available from the corresponding author on reasonable request.

## Abbreviations

AP: Anteroposterior
AUC: Area Under the Curve
BMI: Body Mass Index
CI: Confidence Interval
CLAHE: Contrast-Limited Adaptive Histogram Equalization
CORAL: Consistent Rank Logits
DCA: Decision Curve Analysis
ECE: Expected Calibration Error
FDR: False Discovery Rate
FT-Transformer: Feature Tokenizer + Transformer
Grad-CAM++: Gradient-weighted Class Activation Mapping++
IoU: Intersection over Union
IRB: Institutional Review Board
KL: Kellgren–Lawrence
mAP: Mean Average Precision
OA: Osteoarthritis
OAI: Osteoarthritis Initiative
PACS: Picture Archiving and Communication System
QWK: Quadratic Weighted Kappa
RCGF: Robust Cross-Modal Gated Fusion
ROC: Receiver Operating Characteristic
SD: Standard Deviation
SHAP: SHapley Additive exPlanations
t-SNE: t-distributed Stochastic Neighbor Embedding
TRIPOD + AI: Transparent Reporting of a multivariable prediction model for Individual Prognosis Or Diagnosis with Artificial Intelligence
YOLOv8: You Only Look Once version 8
