# Supplementary material for: Clinically integrated multi-modal transformer framework with cross-modal gated fusion and clinical nomogram for automated Kellgren-Lawrence grading of knee osteoarthritis on x-ray images
Source: BMC Musculoskelet Disord. 2026 Jun 15;27:528. doi: 10.1186/s12891-026-10043-4 (PMC13281478; doi:10.1186/s12891-026-10043-4)
Supplement: Supplementary file 1 — Supplementary Material 1. [file 12891_2026_10043_MOESM1_ESM.docx]

## Supplementary Materials

**Supplementary Table S1.** Classification performance of all eight evaluated architectures across training, internal validation, and external test sets. Training and validation values are mean ± SD across five folds; external test values are point estimates (95% CI). Bold indicates best performance per metric. FDR-corrected p-values are reported for external test comparisons versus the proposed RCGF. MM = multi-modal (image + clinical); UNI = unimodal image-only; CLI = clinical-only. RCGF, Robust Cross-Modal Gated Fusion; QWK, Quadratic Weighted Kappa; ECE, Expected Calibration Error.

| Classifier | Dataset | Accuracy (%) | Sensitivity (%) | Specificity (%) | Precision (%) | F1-score | QWK | AUC | Brier Score | ECE | P-value |
| --- | --- | --- | --- | --- | --- | --- | --- | --- | --- | --- | --- |
| Proposed RCGF (MM) | **Training** | **90.1 ± 0.8** | **90.4 ± 0.9** | **94.2 ± 0.7** | **89.8 ± 0.9** | **0.901 ± 0.009** | **0.901 ± 0.008** | **0.960 ± 0.010** | **0.071 ± 0.006** | **0.038 ± 0.004** | **—** |
|  | Validation | 88.4 ± 1.1 | 89.2 ± 1.4 | 93.1 ± 0.9 | 88.6 ± 1.2 | 0.889 ± 0.012 | 0.884 ± 0.011 | 0.950 ± 0.010 | 0.074 ± 0.007 | 0.041 ± 0.005 | — |
|  | External | 86.3 (83.9–88.5) | 87.1 (84.6–89.3) | 92.4 (90.1–94.3) | 86.5 (84.0–88.8) | 0.870 (0.846–0.891) | 0.900 (0.877–0.921) | 0.930 (0.910–0.950) | 0.072 (0.061–0.084) | 0.039 (0.031–0.048) | ***Ref.*** |
| BioViL-T (UNI) | Training | 84.3 ± 1.3 | 85.1 ± 1.5 | 90.4 ± 1.1 | 83.9 ± 1.3 | 0.846 ± 0.013 | 0.840 ± 0.012 | 0.901 ± 0.018 | 0.091 ± 0.008 | 0.052 ± 0.005 | — |
|  | Validation | 83.6 ± 1.4 | 84.5 ± 1.6 | 89.8 ± 1.2 | 83.2 ± 1.4 | 0.839 ± 0.014 | 0.833 ± 0.013 | 0.891 ± 0.019 | 0.093 ± 0.009 | 0.054 ± 0.006 | — |
|  | External | 82.7 (80.2–84.9) | 83.6 (80.9–85.9) | 89.1 (86.5–91.3) | 82.2 (79.6–84.7) | 0.831 (0.805–0.854) | 0.850 (0.824–0.874) | 0.890 (0.864–0.914) | 0.094 (0.081–0.108) | 0.055 (0.045–0.066) | <0.001 |
| Late-Fusion (MM) | Training | 84.7 ± 1.2 | 85.3 ± 1.4 | 90.8 ± 1.1 | 84.1 ± 1.3 | 0.849 ± 0.013 | 0.843 ± 0.012 | 0.902 ± 0.018 | 0.089 ± 0.008 | 0.051 ± 0.005 | — |
|  | Validation | 83.1 ± 1.5 | 84.2 ± 1.7 | 89.5 ± 1.3 | 82.8 ± 1.5 | 0.835 ± 0.015 | 0.829 ± 0.014 | 0.891 ± 0.020 | 0.092 ± 0.009 | 0.054 ± 0.006 | — |
|  | External | 81.9 (79.4–84.2) | 82.8 (80.1–85.3) | 88.7 (86.2–90.9) | 81.4 (78.8–83.8) | 0.825 (0.799–0.849) | 0.825 (0.799–0.849) | 0.880 (0.854–0.904) | 0.096 (0.083–0.110) | 0.057 (0.047–0.068) | <0.001 |
| MedViT (UNI) | Training | 82.9 ± 1.4 | 83.5 ± 1.6 | 89.1 ± 1.2 | 82.2 ± 1.4 | 0.829 ± 0.014 | 0.823 ± 0.013 | 0.891 ± 0.019 | 0.096 ± 0.009 | 0.057 ± 0.006 | — |
|  | Validation | 81.4 ± 1.6 | 82.3 ± 1.8 | 88.2 ± 1.4 | 80.9 ± 1.6 | 0.815 ± 0.016 | 0.809 ± 0.015 | 0.881 ± 0.021 | 0.099 ± 0.010 | 0.059 ± 0.007 | — |
|  | External | 80.2 (77.6–82.5) | 81.1 (78.4–83.6) | 87.4 (84.9–89.6) | 79.7 (77.1–82.2) | 0.806 (0.779–0.830) | 0.830 (0.803–0.855) | 0.870 (0.843–0.895) | 0.103 (0.089–0.118) | 0.062 (0.051–0.074) | <0.001 |
| ViT-B/16 (UNI) | Training | 80.8 ± 1.5 | 81.5 ± 1.7 | 87.9 ± 1.3 | 80.2 ± 1.5 | 0.810 ± 0.015 | 0.804 ± 0.014 | 0.869 ± 0.022 | 0.109 ± 0.011 | 0.068 ± 0.007 | — |
|  | Validation | 79.4 ± 1.7 | 80.3 ± 1.9 | 87.0 ± 1.5 | 78.9 ± 1.7 | 0.796 ± 0.017 | 0.790 ± 0.016 | 0.859 ± 0.024 | 0.112 ± 0.012 | 0.070 ± 0.008 | — |
|  | External | 79.6 (77.0–82.1) | 80.4 (77.7–83.0) | 87.0 (84.4–89.4) | 79.1 (76.4–81.7) | 0.800 (0.773–0.824) | 0.819 (0.792–0.844) | 0.863 (0.836–0.888) | 0.107 (0.093–0.122) | 0.066 (0.055–0.078) | <0.001 |
| EfficientNet-B7 (UNI) | Training | 80.1 ± 1.6 | 80.8 ± 1.8 | 87.3 ± 1.4 | 79.5 ± 1.6 | 0.803 ± 0.016 | 0.797 ± 0.015 | 0.864 ± 0.023 | 0.112 ± 0.011 | 0.070 ± 0.007 | — |
|  | Validation | 78.7 ± 1.8 | 79.6 ± 2.0 | 86.2 ± 1.6 | 78.1 ± 1.8 | 0.789 ± 0.018 | 0.783 ± 0.017 | 0.853 ± 0.025 | 0.115 ± 0.012 | 0.072 ± 0.008 | — |
|  | External | 78.9 (76.3–81.4) | 79.7 (77.0–82.3) | 86.4 (83.8–88.8) | 78.3 (75.6–80.9) | 0.793 (0.766–0.818) | 0.812 (0.784–0.838) | 0.858 (0.831–0.883) | 0.109 (0.095–0.124) | 0.068 (0.056–0.080) | <0.001 |
| ResNet-50 (UNI) | Training | 77.8 ± 1.9 | 78.4 ± 2.1 | 85.6 ± 1.7 | 77.1 ± 1.9 | 0.779 ± 0.019 | 0.773 ± 0.018 | 0.846 ± 0.026 | 0.121 ± 0.012 | 0.076 ± 0.008 | — |
|  | Validation | 76.2 ± 2.1 | 77.1 ± 2.3 | 84.4 ± 1.9 | 75.6 ± 2.1 | 0.764 ± 0.021 | 0.758 ± 0.019 | 0.836 ± 0.028 | 0.124 ± 0.013 | 0.078 ± 0.009 | — |
|  | External | 76.4 (73.7–79.0) | 77.2 (74.4–79.9) | 84.8 (82.1–87.3) | 75.8 (73.0–78.5) | 0.768 (0.740–0.794) | 0.789 (0.761–0.815) | 0.841 (0.813–0.867) | 0.118 (0.103–0.134) | 0.074 (0.062–0.087) | <0.001 |
| XGBoost-Clinical (CLI) | Training | 72.4 ± 2.0 | 73.1 ± 2.3 | 83.1 ± 1.8 | 71.8 ± 2.0 | 0.724 ± 0.021 | 0.717 ± 0.019 | 0.796 ± 0.028 | 0.141 ± 0.014 | 0.091 ± 0.010 | — |
|  | Validation | 70.5 ± 2.3 | 71.2 ± 2.6 | 81.4 ± 2.1 | 69.8 ± 2.3 | 0.705 ± 0.024 | 0.698 ± 0.022 | 0.781 ± 0.031 | 0.142 ± 0.015 | 0.092 ± 0.011 | — |
|  | External | 69.2 (66.5–71.7) | 69.8 (67.1–72.4) | 80.3 (77.6–82.7) | 68.5 (65.8–71.1) | 0.692 (0.664–0.717) | 0.770 (0.740–0.798) | 0.771 (0.742–0.799) | 0.148 (0.131–0.166) | 0.097 (0.083–0.112) | <0.001 |

**Supplementary Table S2.** Imaging acquisition parameters for each of the three participating centers. SID, source-to-image distance; AP, anteroposterior; DR, digital radiography; CsI, cesium iodide.

| Parameter | Center 1 | Center 2 | Center 3 |
| --- | --- | --- | --- |
| Scanner vendor | Siemens Healthineers (Multix Impact) | GE Healthcare (Discovery XR656) | Philips Healthcare (DigitalDiagnost C90) |
| Detector type | Flat-panel DR (amorphous silicon) | Flat-panel DR (CsI scintillator) | Flat-panel DR (amorphous silicon + CsI) |
| kVp range | 60–70 kVp | 58–72 kVp | 62–68 kVp |
| mAs range | 5–12 mAs | 4–14 mAs | 5–10 mAs |
| Pixel spacing (mm) | 0.143 × 0.143 | 0.148 × 0.148 | 0.139 × 0.139 |
| Image matrix | 2480 × 3072 | 2560 × 3072 | 2448 × 3072 |
| SID (cm) | 115 cm | 110 cm | 120 cm |
| Patient positioning | Weight-bearing AP | Weight-bearing AP | Weight-bearing AP |

**Supplementary Table S3.** Preliminary ablation comparing Swin Transformer backbone variants on the external test set. Swin-Base was selected based on comparable QWK with substantially lower parameter count and inference time. ΔQWK between Swin-Base and Swin-Large = 0.002 (p = 0.41, DeLong's test).

| Architecture | Parameters (M) | External QWK | Inference (ms, A100) | Selected |
| --- | --- | --- | --- | --- |
| Swin-Base | 98.7 | **0.900** | 28 | **✓** |
| Swin-Large | 196.5 | 0.902 | 49 | — |
| SwinV2-Base | 102.1 | 0.899 | 31 | — |
